# Intravenous Arginine Administration Attenuates the Inflammatory Response and Improves Metabolic Profiles in Diet-Induced Obese Mice after Sleeve Gastrectomy

**DOI:** 10.3390/metabo12020153

**Published:** 2022-02-07

**Authors:** Ya-Ling Chen, Ming-Tsan Lin, Wan-Hsuan Wang, Sung-Ling Yeh, Chiu-Li Yeh

**Affiliations:** 1 School of Nutrition and Health Sciences, College of Nutrition, Taipei Medical University, Taipei 11031, Taiwan; ylchen01@tmu.edu.tw (Y.-L.C.); ma07108002@tmu.edu.tw (W.-H.W.); 2 Research Center of Geriatric Nutrition, College of Nutrition, Taipei Medical University, Taipei 11031, Taiwan; 3 Department of Surgery, National Taiwan University Hospital and College of Medicine, National Taiwan University, Taipei 100225, Taiwan; linmt@ntu.edu.tw (M.-T.L.); sangling@tmu.edu.tw (S.-L.Y.); 4 Nutrition Research Center, Taipei Medical University Hospital, Taipei 11031, Taiwan

**Keywords:** macrophage polarization, adipocyte inflammation, hepatic lipid β-oxidation, lipid peroxide

## Abstract

Sleeve gastrectomy (SG) is a bariatric surgery that can effectively reduce weight and improve obesity-associated comorbidities. However, surgical stress intensifies inflammation and imbalanced metabolic profiles. Arginine (Arg) is a nutrient with immunomodulatory and anti-inflammatory properties. This study evaluated the short-term effects of Arg administration on adipocyte inflammation and metabolic alterations in obese mice after SG. Mice were assigned to normal and high-fat diet (HFD) groups. After 16 weeks, the HFD group were divided to sham (SH), SG with saline (SS), or Arg (SA) groups. SS and SA groups were postoperatively injected with saline or Arg via the tail vein and sacrificed at day 1 or 3 after the SG, respectively. Results showed that obesity caused elevated plasma glucose and leptin levels. The SG operation enhanced the expression of inflammatory cytokines and macrophage infiltration in adipose tissues, whereas hepatocyte gene expressions associated with lipid β-oxidation were downregulated. Arg treatment reversed the expressions of β-oxidation-associated genes and reduced lipid peroxide production in the liver. Additionally, adipose tissue expressions of inflammatory chemokines were reduced, while the M2 macrophage marker increased after surgery. The findings suggest that postoperative Arg administration elicited more balanced hepatic lipid metabolism, polarized macrophages toward the anti-inflammatory type, and attenuated adipocyte inflammation shortly after SG.

## 1. Introduction

Obesity is a worldwide epidemic, and the prevalence has dramatically increased over the past two decades in Taiwan [1]. Excessive adipose tissue accumulation and enlarged adipocytes result in macrophage infiltration and persistent inflammation, which may lead to insulin resistance, dysregulated lipid metabolism, and other metabolic dysfunctions [2]. Weight reduction is an effective strategy in attenuating obesity-related complications. A low-caloric diet, behavior modifications, and drug interventions are commonly used strategies; however, these conservative treatments have limited effects on long-term weight control, especially in subjects with morbid obesity [3,4]. Sleeve gastrectomy (SG) is a bariatric surgery that is frequently used and considered to be effective for losing weight and associated comorbidities in morbid obesity [5,6]. However, obesity delays wound healing [7], and surgical stress itself intensifies inflammatory reactions and imbalanced metabolic profiles [8] that need to be corrected in order to improve subsequent outcomes for the long term.

Arginine (Arg) is a semi-essential amino acid and a precursor for nitric oxide (NO) [9]. Numerous studies have confirmed that Arg has properties of immunomodulation and anti-inflammation in catabolic conditions [10,11,12]. Previous studies found that the availability of Arg is impaired in diabetic-obese rats, and that impairment was associated with increased inflammation and oxidative stress [13,14]. Arg supplementation may be useful in enhancing Arg availability and attenuating inflammation in obesity and catabolic conditions. On the other hand, Arg was reported to reduce central adiposity [15]. Animal studies also showed that Arg supplementation reduced white fat gain and improved metabolic profiles in diet-induced obesity [16,17]. Although Arg seems to provide beneficial effects on obesity, to the best of our knowledge, no study has investigated the impacts of Arg on inflammatory reactions and metabolic alterations in relation to bariatric surgery. In this study, we used a diet-induced obese mouse model and performed SG surgery to mimic human operation on morbidly obese patients. Systemic and adipocyte inflammation as well as metabolic profile changes were reported in both obesity and surgical stress [2,7,8]. Besides, obesity-induced liver steatosis can potentially impair hepatic functions and dysregulate the balance of lipid metabolism [18,19]. This study administered Arg intravenously after an SG in obese mice. Indicators of inflammatory responses and genes associated with fatty acid (FA) metabolism were analyzed 1 and 3 days after gastrectomy. We hypothesized that Arg administration can improve metabolic profiles and resolve inflammation in obese mice shortly after the bariatric surgery.

## 2. Results

### 2.1. Body Weights (BWs), Epididymal Fat Weights, and Blood Glucose Changes during the Intraperitoneal Glucose Tolerance Test (IPGTT)

**Figure 1:**
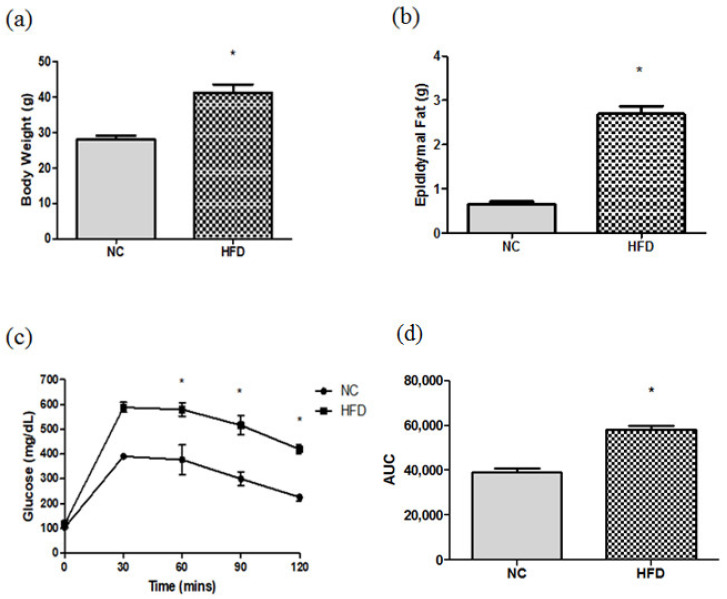
(**a**) Body weights, (**b**) epididymal fat weights, (**c**) dynamic changes in blood glucose levels, and (**d**) area under the curve (AUC) of mice fed the chow diet and high-fat diet (HFD) during the intraperitoneal glucose tolerance test (IPGTT). NC: normal control; *n* = 6 for each group. Data are presented as the mean ± SEM. Differences between groups were analyzed by an unpaired t-test. * *p* < 0.05.

High-fat diet (HFD) feeding resulted in significant BWs, epididymal fat gain, and higher blood glucose levels at 60, 90, and 120 min after glucose loading. The area under the curve (AUC) was significantly higher in the HFD-fed group than that fed with a chow diet (Figure 1). 

### 2.2. Body Weight Change and Epidydymal Weights after the Gastrectomy

There were no differences in initial body weights before the gastrectomy. Body weight loss was found in all the experimental groups after the surgery and the weight changes were even greater at day 3 than day 1. There were no differences in body weight changes and epididymal fat weights between the SS and SA groups at each time point (Table 1).

### 2.3. Plasma Levels of Biochemical Parameters

The obese sham group (SH) had higher total cholesterol (TC) and glucose levels than the normal control (NC) group. SG surgery resulted in higher aspartate aminotransferase (AST), alanine aminotransferase (ALT), and insulin levels, while high-density lipoprotein-cholesterol (HDL-C) and glucose levels were lower than those in the SH group. Different from the saline group with SG surgery (SS) groups, the Arg group with SG surgery (SA) groups exhibited comparable AST levels to the SH group. Additionally, levels of ALT, triglycerides (TGs), and TC were lower, while HDL-C levels were higher than those in the SS group on day 1 and/or day 3 after surgery (Table 2).

### 2.4. Plasma Nitric Oxide (NO) and Adipokine Levels

The SH group had lower adiponectin levels than the NC group. There were no differences in NO or leptin levels between the NC and SH groups. Concentrations of leptin increased and those of adiponectin decreased after SG surgery. Compared to the SS groups, Arg administration increased NO and adiponectin on day 3 and reduced leptin levels on day 1 after the surgery (Table 3).

### 2.5. Plasma Amino Acid Concentrations

**Figure 2:**
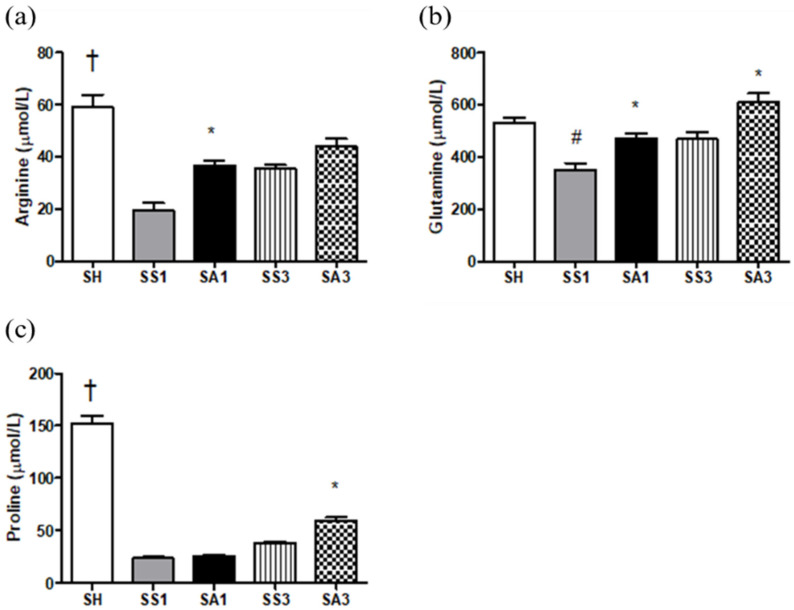
Plasma amino acid concentrations among the experimental groups. (**a**) Arginine, (**b**) glutamine, and (**c**) proline. Data are presented as the mean ± SEM. SH, sham group without a sleeve gastrectomy (SG); SS1 and SS3, saline groups sacrificed on day 1 or 3 after the SG; SA1 and SA3, arginine groups sacrificed on day 1 or 3 after the SG. A two-way analysis of variance (ANOVA) with the Bonferroni post-hoc test was used to analyze differences among the SH and gastrectomy groups. † Significantly differs from the gastrectomy groups. # Significantly differs from the SH group. * Significantly differs from the SS group at the same time point (*p* < 0.05).

Compared to the SH group, Arg, proline, and glutamine levels were significantly lower after the SG. Although Arg administration could not restore Arg and proline to levels comparable to the SH group, Arg, proline, and glutamine levels increased after Arg treatment (Figure 2).

### 2.6. Inflammatory Cytokine Levels in Peritoneal Lavage Fluid (PLF)

**Figure 3:**
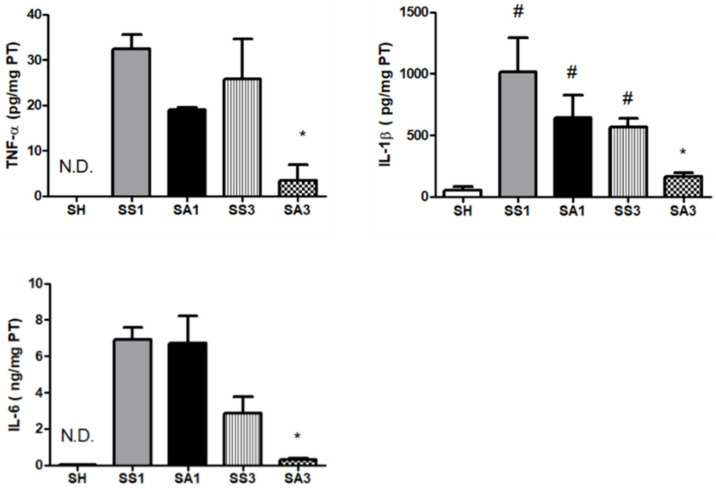
Concentrations of inflammatory cytokines in peritoneal lavage fluid (PLF). IL, interleukin; TNF, tumor necrosis factor; SH, sham group without a sleeve gastrectomy (SG) (*n* = 8); N.D.: Not Detected; SS1 and SS3, saline groups sacrificed on day 1 or 3 after the SG; SA1 and SA3, arginine groups sacrificed on day 1 or 3 after the SG. *n* = 10 for each SG group. Values are presented as the mean ± SEM. A two-way analysis of variance (ANOVA) with the Bonferroni post-hoc test was used to analyze differences among the SH and gastrectomy groups. # Significantly differs from the SH group. * Significantly differs from the SS group at the same time point (*p* < 0.05).

Tumor necrosis factor (TNF)-α and interleukin (IL)-6 levels in the SH group were not detectable. SG surgery resulted in significantly increased TNF-α, IL-1β, and IL-6 levels. Compared to the SS group, the SA group exhibited lower TNF-α, IL-1β, and IL-6 concentrations on day 3 after the gastrectomy (Figure 3).

### 2.7. Messenger (m)RNA Expression of Peroxisome Proliferator-Activated Receptor (PPAR)-γ in Adipose Tissues

**Figure 4:**
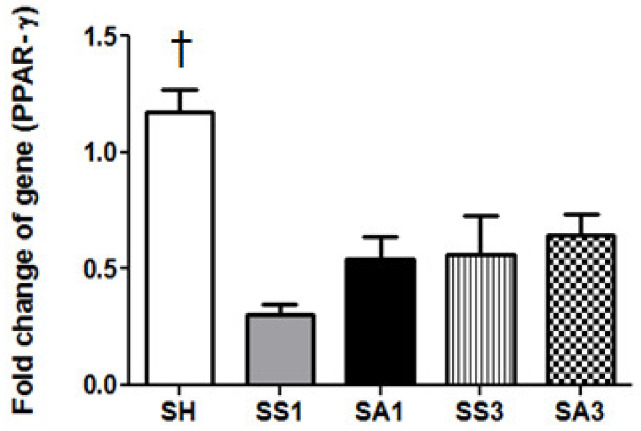
Messenger RNA expressions of PPAR-γ in epididymal fat tissues. SH, sham group without a sleeve gastrectomy (SG) (*n* = 8); SS1 and SS3, saline groups sacrificed on day 1 or 3 after the SG; SA1 and SA3, arginine groups sacrificed on day 1 or 3 after the SG. *n* = 10 for each SG group. Values are presented as the mean ± SEM. A two-way analysis of variance (ANOVA) with the Bonferroni post-hoc test was used to analyze differences among the SH and gastrectomy groups. † Significantly differs from the gastrectomy groups (*p* < 0.05).

The SH group had the highest PPAR-γ expression among all groups. There were no differences in the expression of PPAR-γ among the SS and SA groups at each time point (Figure 4).

### 2.8. mRNA Expressions of Inflammatory Mediators and Macrophage Infiltration Markers in Adipose Tissues

**Figure 5:**
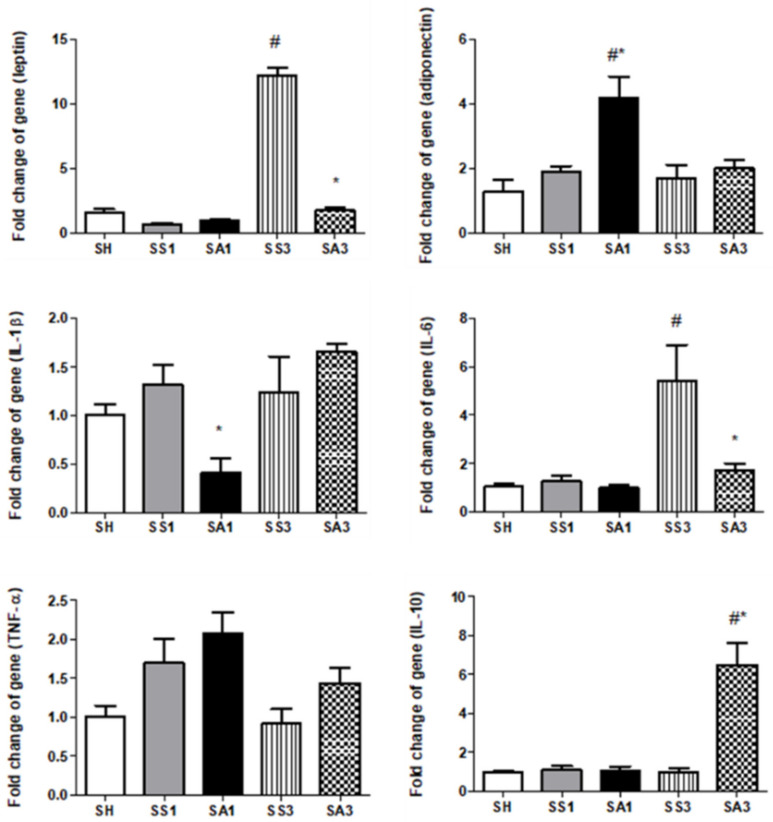
Messenger RNA expressions of pro- and anti-inflammatory mediators in epididymal fat tissues. IL, interleukin; TNF, tumor necrosis factor; SH, sham group without a sleeve gastrectomy (SG) (*n* = 8); SS1 and SS3, saline groups sacrificed on day 1 or 3 after the SG; SA1 and SA3, arginine groups sacrificed on day 1 or 3 after the SG. *n* = 10 for each SG group. Values are presented as the mean ± SEM. A two-way analysis of variance (ANOVA) with the Bonferroni post-hoc test was used to analyze differences among the SH and gastrectomy groups. # Significantly differs from the SH group. * Significantly differs from the SS group at the same time point (*p* < 0.05).

**Figure 6:**
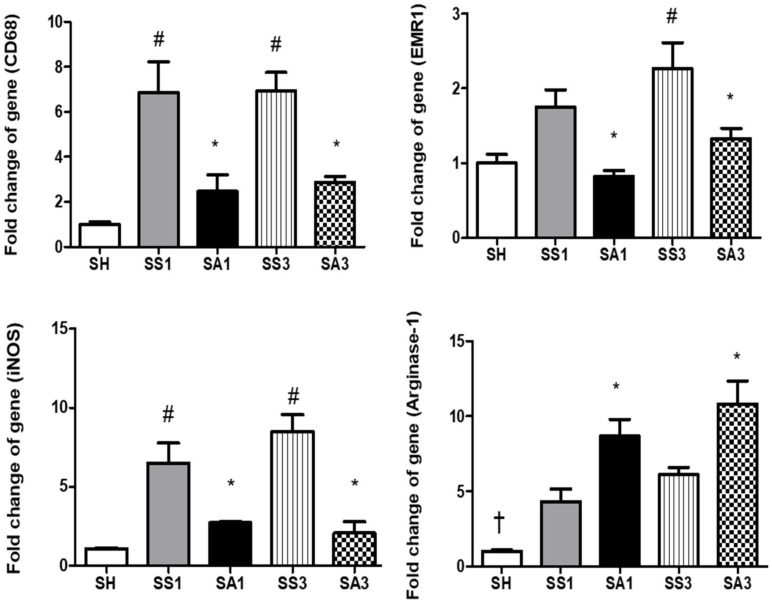
Messenger RNA expressions of macrophage infiltration markers in epididymal fat tissues. CD68, cluster of differentiation 68; EMR1, epidermal growth factor-like module-containing mucin-like hormone receptor-like 1; iNOS, inducible nitric oxide synthase. SH, sham group without a sleeve gastrectomy (SG) (*n* = 8); SS1 and SS3, saline groups sacrificed on day 1 or 3 after the SG; SA1 and SA3, arginine groups sacrificed on day 1 or 3 after the SG. *n* = 10 for each SG group. Values are presented as the mean ± SEM. A two-way analysis of variance (ANOVA) with the Bonferroni post-hoc test was used to analyze differences among the SH and gastrectomy groups. + Significantly differs from the gastrectomy groups. # Significantly differs from the SH group. * Significantly differs from the SS group at the same time point (*p* < 0.05).

Pronounced increments in leptin and IL-6 were noted on day 3 after the SG. Compared to the SS group, Arg treatment resulted in significantly lower leptin, IL-1β, and IL-6 expressions, while adiponectin and IL-10 were higher on either day 1 or 3 postoperatively (Figure 5). mRNA expression levels of cluster of differentiation (CD)68 on days 1 and 3 and epidermal growth factor-like module-containing mucin-like hormone receptor-like (EMR)1 on day 3 after the SG were significantly higher than those of the SH group. Both inducible nitric oxide synthase (iNOS) and arginase-1 expressions were elevated after the gastrectomy. Compared to the SS groups, the SA groups showed significantly lower CD68, EMR-1, and iNOS and higher arginase-1 expressions after the SG (Figure 6).

### 2.9. Lipid Metabolism-Related Gene Expressions in the Liver

**Figure 7:**
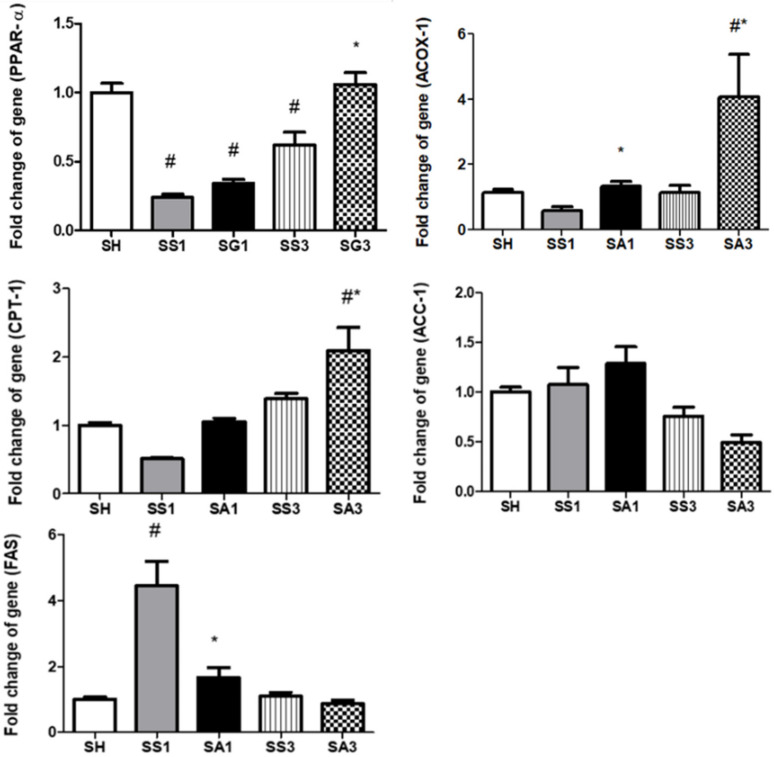
Messenger RNA expressions of lipid metabolism-associated genes in liver tissues. ACC, acetyl-CoA carboxylase; ACOX, acyl-CoA oxidase; CPT, carnitine palmitoyltransferase; FAS, fatty acid synthase; PPAR, peroxisome proliferator-activated receptor. SH, sham group without a sleeve gastrectomy (SG) (*n* = 8); SS1 and SS3, saline groups sacrificed on day 1 or 3 after the SG; SA1 and SA3, arginine groups sacrificed on day 1 or 3 after the SG. *n* = 10 for each SG group. Values are presented as the mean ± SEM. A two-way analysis of variance (ANOVA) with the Bonferroni post-hoc test was used to analyze differences among the SH and gastrectomy groups. # Significantly differs from the SH group. * Significantly differs from the SS group at the same time point (*p* < 0.05).

Compared to the SH group, PPAR-α expression decreased whereas fatty acid synthase (FAS) and acyl-CoA oxidase (ACOX)-1 expressions increased after the gastrectomy. Groups treated with Arg exhibited higher ACOX-1 expressions on both days 1 and 3, and PPAR-α and carnitine palmitoyltransferase (CPT)-1 expressions on day 3 after the SG; in contrast, FAS expression was lower than those expressed in the SS groups (Figure 7).

### 2.10. Lipid Peroxide Concentrations in Liver Tissues

**Figure 8:**
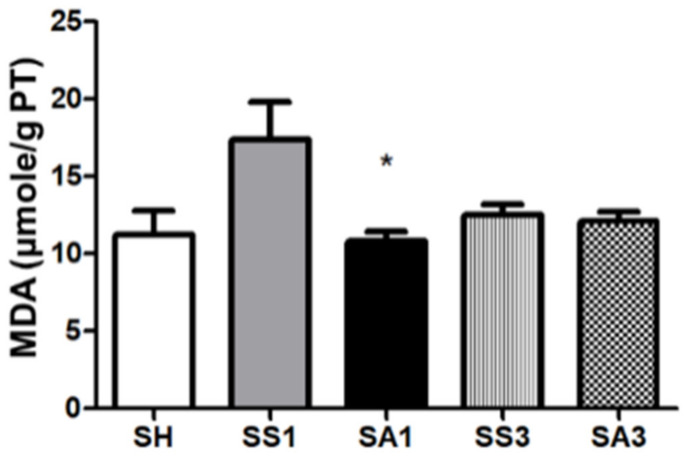
Lipid peroxide malondialdehyde (MDA) concentrations in liver tissues. SH, sham group without a sleeve gastrectomy (SG) (*n* = 8); SS1 and SS3, saline groups sacrificed on day 1 or 3 after the SG; SA1 and SA3, arginine groups sacrificed on day 1 or 3 after the SG. *n* = 10 for each SG group. Values are presented as the mean ± SEM. A two-way analysis of variance (ANOVA) with the Bonferroni post-hoc test was used to analyze differences among the SH and gastrectomy groups. * Significantly differs from the SS group at the same time point (*p* < 0.05).

The SG surgery tended to produce higher malondialdehyde (MDA) levels; however, there was no significant difference between the SH and SS groups. The SA group had a lower MDA concentration than the SS group on day 1 after the gastrectomy (Figure 8).

## 3. Discussion

HFD-induced excessive calorie intake is a common cause of obesity. In this study, we used the C57BL/6 mouse strain, which is thought to be suitable for obesity research [20]. Heavier epididymal adipose tissues, hyperlipidemia, and hyperglycemia with an impaired response to glucose loading were observed after feeding the HFD. Our findings were similar to a former report on mice with diet-induced obesity [20], and these physiological changes were consistent with those that occur in human obesity. As far as we know, there is no study investigating the influences of intravenous Arg administration on adipocyte inflammatory responses and metabolic profile alterations after bariatric surgery. The main findings of this study revealed that Arg treatment attenuated adipose tissue inflammation and elicited a more balanced metabolic profile in obese mice shortly after the SG.

Adipose tissues are multifunctional. In addition to fat storage, several adipokines are secreted which participate in acute-phase responses, vascular homeostasis, balancing pro- and anti-inflammation, and lipid metabolism, etc. [21]. Adiponectin and leptin are the most familiar adipokines participating in modulating immune responses. Adiponectin has an anti-inflammatory property, while leptin enhances inflammatory responses [21,22]. In this study, we found that compared to the sham operation, an SG promoted expressions of leptin and IL-6 in adipose tissues, and plasma leptin levels and secretion of inflammatory cytokines in the PLF were also elevated. We also noted that CD68 and EMR-1 expressions were upregulated after the SG. CD68 and EMR-1 are macrophage markers expressed on inflamed tissues [23,24]. These findings indicated that macrophage infiltration increased, and inflammation was exacerbated in adipose tissues after SG surgery.

The liver is one of the organs that participates in lipid metabolism. In this study, we analyzed several genes responsible for lipid synthesis and oxidation. Acetyl-CoA carboxylase (ACC) and FAS are related to lipogenesis, while ACOX-1 and CPT1 are involved in β-oxidation [25]. PPARs are nuclear receptors that maintain the metabolic homeostasis by controlling numerous genes involved in inflammation and lipid and glucose metabolism [26,27]. PPAR-α is mainly expressed by tissues, such as the liver, that have high rates of β-oxidation [27]. The findings of our study showed that an SG resulted in lower PPAR-α and ACOX-1 gene expressions; in parallel, the FAS gene was upregulated, indicating that lipogenesis was enhanced while β-oxidation was suppressed after the operation.

In this study, we found that compared to obese mice injected with saline, intravenous Arg administration after an SG exhibited several favorable effects. First, Arg administration restored the reduced plasma Arg levels, which may have improved the availability of Arg and NO after surgery. The increased inflammation and oxidative stress in obesity with metabolic disorders are thought to be associated with the reduced availability of Arg [13]. In this study, we noted decreased plasma Arg levels after the SG, and Arg administration increased Arg levels. In addition, elevated glutamine and proline levels were observed. In addition to the precursor of NO, Arg is also a substrate for proline synthesis via the urea cycle. Proline is an essential constituent of collagen [28]. Glutamine is a specific nutrient with anti-oxidative and anti-inflammatory properties [29]. The amino acid profiles showed that the Arg-treated group may have beneficial effects on alleviating inflammation and metabolic dysregulation. Second, Arg administration alleviated adipocyte inflammation and macrophage infiltration after the SG. Downregulated leptin, IL-1β, and IL-6, and upregulated adiponectin and IL-10 expressions suggest that the inflammatory response was suppressed. Additionally, reduced CD68 and EMR-1 levels indicated that the infiltration of macrophages into the adipose tissue was reduced in the Arg-treated groups. A previous study reported that Arg modulates immune responses during infections partly by manipulating the polarization of M1/M2 macrophages [30]. Infiltration of M1-type macrophages results in inflammation and tissue damage. In contrast, the M2 type attenuates inflammation and enhances tissue repair [31]. The M1 type is characterized by iNOS expression, while the M2 type expresses the enzyme arginase-1 [30]. We noted that with decreased iNOS expression, expression of arginase-1 increased when treated with Arg. These findings suggest that the pro-inflammatory M1 macrophages were polarized toward the anti-inflammatory M2 type, which may attenuate inflammation in adipose tissues. Reduced inflammatory cytokine production in the PLF also suggested that Arg treatment mitigated inflammation in the abdomen. Third, Arg administration to obese mice elicited a more balanced lipid metabolism after the SG. Upregulation of PPAR-α, ACOX-1, and CPT-1 accompanied by downregulation of FAS expression indicated that lipid metabolism had reversed from lipogenesis toward FA oxidation. PPAR-α activation was reported to stimulate enzymes involved in β-oxidation and raise HDL-C levels [27,32]. Consistent with the findings described above, our results also showed a more favorable plasma lipid profile as elevated HDL-C and decreased TC and TGs were noted in the Arg-treated group. There is a close association between inflammation and metabolic dysregulation [33]. In this study, we found that plasma liver injury markers ALT and AST and MDA levels were reduced, suggesting that the oxidative stress and damage to hepatocytes were attenuated when Arg was administered after the SG. Mitigating inflammation and oxidative stress may contribute to improvements in liver function and metabolic profiles.

Previous studies found that SG surgery alleviates postoperative comorbidities [5]. We also found that glucose levels were reduced after the SG surgery. Although insulin concentrations were elevated on day 1, they had dropped to levels comparable to the sham group by day 3. These results suggest that glucose intolerance and insulin resistance had improved after surgery. In this study, we did not find an influence of Arg on glucose tolerance. Additionally, Arg did not show any effect on enhancing body and fat weight loss. PPAR-γ is the master regulator of adipogenesis [34]. The PPAR-γ expression in the adipose tissue did not differ between the groups with or without Arg, suggesting that Arg may not be potent enough to influence the adipogenic differentiation shortly after the SG. Since only three days after SG were examined, the long-term impacts of Arg on obesity with bariatric surgery require further investigation. There is a limitation in this study in that only gene expressions were measured in liver and adipose tissues, although mRNA levels have considerable evidence that has been presented in many experiments. Protein analysis may further strengthen the findings observed in this study.

In conclusion, this is the first study to evaluate the influences of intravenous Arg treatment on metabolic profiles and inflammatory responses in obese mice after an SG. The findings revealed that obese mice with Arg treatment after bariatric surgery exhibited increased plasma Arg levels, enhanced hepatic lipid oxidation, and improved plasma lipid profiles. Additionally, macrophage infiltration was reduced, and adipose tissue inflammation was alleviated after the SG. These findings suggest that Arg has the potential to improve metabolic profiles and alleviate inflammation immediately after bariatric surgery, which may subsequently have long-term benefits.

## 4. Materials and Methods

### 4.1. Animals

Male C57BL/6 mice aged 5 weeks and weighing 18~20 g were housed in the Laboratory Animal Center at Taipei Medical University (TMU, Taipei, Taiwan). The rooms of the animal center were controlled at 21 ± 2 °C and 50~55% humidity, with a 12 h light–dark cycle. Water and rodent chow diet (Purina no. 5001, Fort Worth, TX, USA) were fed ad libitum. The Guide for the Care and Use of Laboratory Animals (National Research Council, 1996) was used to guide the animal care and the experimental protocols were approved by the Animal Care and Use Committee at TMU (LAC-2019-0183).

### 4.2. Experimental Grouping and Design

After an acclimation period of 1 week, mice with comparable weights were assigned to a NC (*n* = 6) group and a high-fat (HF, *n* = 54) group. Rodent chow diet was fed to the mice in the NC group, while a HFD was provided to the HF group for 16 weeks. The HFD consisted of 60% of kilocalories as fat. The diet composition was provided by a commercial company (Research Diets, New Brunswick, NJ, USA), and its contents are provided in Table 4. An IPGTT was performed in mice of the NC and HF groups (*n* = 6 for each group) at 15 weeks after feeding the respective diets. After IPGTT, the mice were sacrificed and the epididymal fats were weighed. The remaining mice in the HF group (*n* = 48) were kept on the HFD until the end of 16 weeks. Then, mice in the HF group were divided into a sham group (SH, *n* = 8), gastrectomy group with saline injection (SS, *n* = 20), and gastrectomy group with Arg administration (SA, *n* = 20). A laparotomy without a gastrectomy was carried out on mice in the SH group. The SS and SA groups underwent the SG surgery. The operative procedure of SG was referenced from a mouse model of bariatric surgery [35] and modified by our laboratory, as described previously. All the surgeries were carried out by the same technician. After the operation, the SS and SA groups were further divided into two subgroups, respectively, according to the schedule of sacrifice on day 1 (SS1 and SA1) or day 3 (SS3 and SA3), with 10 mice in each group. The SS1 group was intravenously injected with saline, while the SA1 group was provided a single dose of Arg (300 mg/kg BW) via the tail vein 1 h after SG surgery. Mice in the SS3 and SA3 groups were re-injected with the same dose of saline or Arg at day 2 after the operation. The dosage of Arg used in this study was shown to modulate immune responses and attenuate inflammation under catabolic conditions [11,36]. At the end of day 1 or 3, mice were euthanized by cardiac puncture according to the respective grouping. Plasma were separated from blood samples and PLF was obtained by irrigating with saline. BWs were recorded. The plasma, PLF, and epididymal adipose and liver tissues were collected and frozen at −80 °C for further analysis.

### 4.3. The IP Glucose Tolerance Test (IPGTT)

After feeding the diets for 15 weeks, animals were starved for 12 h. Then, a 20% glucose solution was administered (2 g glucose/kg BW) via an IP injection. Subsequently, blood glucose levels were measured at 0, 30, 60, 90, and 120 min after the glucose load. The starved blood and blood samples obtained from different time points after glucose loading were drawn from a tail vein. The AUC was calculated according to dynamic changes in blood glucose after the IPGTT. Blood glucose concentrations were analyzed using a strip-operated blood glucose sensor (OneTouch Ultra, Inverness Medical, Uxbridge, UK).

### 4.4. Measurements of Plasma Biochemical Markers and Adipokines

Biochemical markers, including AST, ALT, HDL-C, and LDL-C, were analyzed with a VetTest^®^ Chemistry Analyzer (IDEXX Laboratories, Westbrook, MN, USA). TG, TC, and glucose were analyzed by colorimetric methods (Randox, Antrim, Ireland). Insulin, leptin, and adiponectin were measured by enzyme-linked immunosorbent assay (ELISA) kits. NO concentrations were analyzed using a commercial assay kit (R&D Systems, Minneapolis, MN, USA). All procedures followed instructions provided by the manufacturer.

### 4.5. Plasma Concentrations of Amino Acids

Samples were prepared according to the provided instructions (Waters AccQTag derivatization kit, Manchester, UK) and were applied to ultra-performance liquid chromatography (UPLC) for separation (ACQUITY UPLC system, Waters, Manchester, UK). A mass spectrometer (Xevo TQ-XS, Waters) was used for monitoring. The respective amino acid levels were analyzed by Waters MassLynx 4.2 software and quantified by Waters TargetLynx application manager (Waters, Manchester, UK).

### 4.6. Inflammatory Cytokine Levels in PLF

IL-1β, IL-6, and TNF-α were measured by a commercial ELISA kit (R&D Systems). Levels of cytokines were based on milligrams of protein. Protein concentrations in PLF were analyzed by the Bradford protein assay kit (Bio-Rad, Richmond, CA, USA). The procedures follow the instructions provided by the manufacturer.

### 4.7. mRNA Extraction and a Real-Time Reverse-Transcription (RT) Quantitative Polymerase Chain Reaction (qPCR) Analysis

Homogenized epididymal adipose tissues and liver tissues were used. Total RNA was obtained by commercial reagent and the RNA levels were quantified by a spectrophotometer. Quantified RNA was used to synthesize complementary (c)DNA. The cDNA was amplified by a real-time RT-PCR by the QuantStudio™ 1 Real-Time PCR System (Applied Biosystems, Foster City, CA, USA) to obtain mRNA expressions. SYBR Green I was used as the detection format. Genes analyzed in epididymal adipose tissues included PPAR-γ, inflammatory-associated mediators (IL-1β, IL-6, and TNF-α), an anti-inflammatory cytokine (IL-10), adipokines (leptin and adiponectin), macrophage infiltration markers (CD68, EMR1), and polarization markers (arginase-1, iNOS). Lipid metabolism-associated genes measured in the liver included PPAR-α, ACC, ACOX-1, CPT-1, and FAS (Table 5). Primers were purchased from Mission Biotech (Taipei, Taiwan) based on deposited cDNA sequences (GenBank database, NCBI). The amplification procedures followed the previous experiment performed in [37]. The mRNA expression levels were quantified and calculated by cycle threshold (CT) values. Mouse glyceraldehyde 3-phosphate dehydrogenase (GAPDH) was used to normalize the values presented [38].

### 4.8. Analysis of Thiobarbituric Acid-Reactive Substances (TBARS) in the Liver

Liver tissues were homogenized in reagents containing protease, phosphatase inhibitor (ThermoFisher Scientific, Waltham, MA, USA), and tissue protein extraction reagent (T-PER™, ThermoFisher Scientific, Waltham, MA, USA) at 4 °C. Homogenates were centrifuged to obtain the supernatants that were used for the TBARS analysis (Cayman, Ann Arbor, MI, USA). The TBARS consisted mainly of MDA. TBARS levels were determined at an optical density of 530~540 nm. Concentrations of TBARS are expressed as μmole/g protein. Protein levels were analyzed using the Bradford protein assay kit (Bio-Rad, Hercules, CA, USA).

### 4.9. Statistical Analyses

Data are expressed as the mean ± standard error of the mean (SEM). All analyses were conducted using GraphPad Prism 5 (GraphPad Software version 5, La Jolla, CA, USA). Differences between the NC and HF groups were analyzed by an unpaired t-test. The differences among the SH and gastrectomy groups were analyzed by a two-way analysis of variance (ANOVA) with the Bonferroni post-hoc test. A *p* < 0.05 was considered statistically significant.

## Figures and Tables

**Table 1:** Body weights (BW), weight changes, and epididymal fat weights of the experimental groups before and after the sleeve gastrectomy (SG).

Weights	SS1	SA1	SS3	SA3
BW (g)				
Before SG	41.7 ± 2.6	40.2 ± 2.3	39.5 ± 0.6	39.1 ± 0.9
After SG	39.1 ± 2.4	37.4 ± 1.9	34.9 ± 1.3	34.8 ± 1.5
BW change (g)	−2.65 ± 0.48	−2.82 ± 0.39	−4.78 ± 1.07	−4.17 ± 0.75
Epididymal fat (g)	2.54 ± 0.56	2.65 ± 0.51	2.04 ± 0.35	1.84 ± 0.25

Data are presented as the mean ± SEM. SS1 and SS3, saline groups sacrificed on day 1 or 3 after the SG; SA1 and SA3, arginine groups sacrificed on day 1 or 3 after the SG. Differences between the SS and SA groups at the same time point were analyzed by an unpaired t-test.

**Table 2:** Plasma concentrations of biochemical parameters among the experimental groups.

Parameters	NC	SH	SS1	SA1	SS3	SA3
AST (UL)	54.5 ± 7.8	100.2 ± 23.6	224.6 ± 85.3 ^#^	171.4 ± 68.8	237.8 ± 55.2 ^#^	135.4 ± 37.7
ALT (U/L)	12.1 ± 1.0	31.5 ± 14.1	389.7 ± 122.6 ^#^	188.6 ± 48 ^#,^*	171.8 ± 62.5 ^#^	27.1 ± 8.7 *
TGs (mg/dL)	65.4 ± 18.1	60.3 ± 14.6	45.6 ± 12.8	37.4 ± 15.1 ^#^	89.4 ± 9.2 ^#^	60.5 ± 5.1 *
TC (mg/dL)	103.5 ± 8.8	125.3 ± 13.2 ^†^	107.2 ± 18.3	78.4 ± 3.4 ^#,^*	142.1 ± 33.8	101.9 ± 25.2
LDL-C (mg/dL)	14.5 ± 2.1	24.0 ± 6.6 ^†^	20.5 ± 4.1	21.7 ± 2.9	35.8 ± 9.1 ^#^	35.1 ± 7.4 ^#^
HDL-C (mg/dL)	84.3 ± 8.6	95.3 ± 5.4	71.8 ± 10.8 ^#^	76.1 ± 9.7 ^#^	70.7 ± 11.1 ^#^	83.8 ± 7.3 *
Glucose (mg/dL)	110.2 ± 6.1	204.4 ± 11.4 ^†^	103.7 ± 9.1 ^#^	103.8 ± 9.7 ^#^	73.4 ± 14.6 ^#^	87.37 ± 24.9 ^#^
Insulin (µIU/dL)	208.3 ± 63.3	202.7 ± 89.2	537.9 ± 93.5 ^#^	592.6 ± 98.1 ^#^	218.9 ± 35.9	207.6 ± 49.6

Data are presented as the mean ± SEM. Abbreviations: AST, aspartate aminotransferase; ALT, alanine aminotransferase; TGs, triglycerides; TC, total cholesterol; HDL-C, high-density lipoprotein-cholesterol; LDL-C, low-density lipoprotein-cholesterol; NC, normal control; SH, sham group without a sleeve gastrectomy (SG); SS1 and SS3, saline groups sacrificed on day 1 or 3 after the SG; SA1 and SA3, arginine groups sacrificed on day 1 or 3 after the SG. Differences between the NC and SH groups were analyzed by an unpaired t-test. A two-way analysis of variance (ANOVA) with the Bonferroni post-hoc test was used to analyze differences among the SH and gastrectomy groups. ^†^ Significantly differs from the NC group. ^#^ Significantly differs from the SH group. * Significantly differs from the SS group at the same time point (*p* < 0.05).

**Table 3:** Plasma nitric oxide (NO) and adipokines among the experimental groups.

Parameters	NC	SH	SS1	SA1	SS3	SA3
NO (µmol/dL)	42.2 ± 7.2	34.5 ± 8.1	45.7 ± 12.3	48.1 ± 10.5	22.3 ± 4.9	40.3 ± 2.7 *
Leptin (ng/dL)	11.1 ± 5.7	34.6 ± 18.4	406.2 ± 101.2 ^#^	302.7 ± 71.8 ^#,^*	31.2 ± 15.1	32.6 ± 12.7
Adiponectin (µg/dL)	11.2 ± 0.6	9.2 ± 1.4 ^†^	3.9 ± 0.5 ^#^	3.7 ± 0.3 ^#^	4.3 ± 1.3 ^#^	6.1 ± 0.9 ^#,^*

Data are presented as the mean ± SEM. NC, normal control; SH, sham group without a sleeve gastrectomy (SG); SS1 and SS3, saline groups sacrificed on day 1 or 3 after the SG; SA1 and SA3, arginine groups sacrificed on day 1 or 3 after the SG. Differences between the NC and SH groups were analyzed by an unpaired t-test. A two-way analysis of variance (ANOVA) with the Bonferroni post-hoc test was used to analyze differences among the SH and gastrectomy groups. ^†^ Significantly differs from the NC group. ^#^ Significantly differs from the SH group. * Significantly differs from the SS group at the same time point (*p* < 0.05).

**Table 4:** Compositions of the experimental diets.

Ingredient	60% High-Fat Diet	Normal Diet
Casein, lactic (g)	258.45	189.56
Cysteine, L (g)	3.88	2.84
Corn starch (g)	-	521.30
Maltodextrin (g)	161.53	142.17
Sucrose (g)	94.08	3.79
Cellulose (g)	64.61	47.39
Lard (g)	316.60	18.96
Soybean oil (g)	32.31	23.70
Mineral mix ^1^ (g)	64.61	47.39
Choline bitartrate (g)	2.58	1.90
Vitamin mix ^2^ (g)	1.29	0.95
Dye (g)	0.06	0.06
Total (g)	1000	1000
Protein/Fat/Carbohydrate (%)	20/60/20	20/10/70
Energy density (kcal/g)	5.21	3.82

^1^ The composition of the mineral mixture was as follows (g/1000 g): potassium citrate, 330; calcium phosphate, 260; calcium carbonate, 110; sodium chloride, 51.8; magnesium sulfate, 51.52; magnesium oxide, 8.38; ferric citrate 4.2; manganese carbohydrate hydrate, 2.45; zinc carbonate, 1.12; chromium potassium sulfate, 0.39; copper carbonate, 0.21; ammonium molybdate tetrahydrate, 0.06; sodium fluoride, 0.04; sodium selenite, 0.01; potassium iodate, 0.01. ^2^ The composition of the vitamin mixture was as follows (g/100 g): vitamin E acetate, 10; niacin, 3; biotin (1%), 2; pantothenic acid, 1.6; vitamin D3, 1; vitamin B12, 1; vitamin A acetate, 0.8; pyridoxine HCl, 0.7; riboflavin, 0.6; thiamine HCl, 0.6; folic acid, 0.2; menadione sodium bisulfite, 0.08. The compositions of the vitamin mixture were as follows (mg/g): DL-α-tocopherol acetate, 20; nicotinic acid, 3; retinyl palmitate, 1.6; calcium pantothenate, 1.6; pyridoxine hydrochloride, 0.7; thiamin hydrochloride, 0.6; riboflavin, 0.6; cholecalciferol, 0.25; D-biotin, 0.05; menaquinone, 0.005; cyanocobalamin, 0.001.

**Table 5:** Sequences of oligonucleotide primers used in the PCR amplification.

Gene Name	Accession No.	5′→3′ Primer Sequence
ACC-1	XM_036156218.1	F: ATGGGCGGAATGGTCTCTTTC R: TGGGGACCTTGTCTTCATCAT
ACOX-1	NM_001377522.1	F: GGATGGTAGTCCGGAGAACA R: AGTCTGGATCGTTCAGAATCAAG
Adiponectin	NM_009605.5	F: ATCTGGAGGTGGGAGACCAA R: GGGCTATGGGTAGTTGCAGT
Arginase-1	AH011507.2	F: AGCAGAAGGCTTTGTCAGCA R: ACCCAAAGTGGCACAACTCA
CD68	NM_001291058.1	F: TGTTCAGCTCCAAGCCCAAA R: ACTCGGGCTCTGATGTAGGT
CPT-1	NM_013495.2	F: GAGCCAGACCTTGAAGTAACG R: GAGACAGACACCATCCAACAC
EMR1	U66889.1	F: ACCTTGTGGTCCTAACTCAGTC R: ACAAAGCCTGGTTGACAGGTA
FAS	NM_007988.3	F: GGAGGTGGTGATAGCCGGTAT R: TGGGTAATCCATAGAGCCCAG
IL-1β	NM_008361.4	F: TGCCACCTTTTGACAGTGATG R: ATGTGCTGCTGCGAGATTTG
IL-6	NM_031168.2	F: TCCTACCCCAACTTCCAATGCTC R: TTGGATGGTCTTGGTCCTTAGCC
IL-10	M37897.1	F: AGGCGCTGTCATCGATTTCT R: ATGGCCTTGTAGACACCTTGG
iNOS	U58677.1	F: ACTAGGGCACCTCCATCACT R: TAATGGGGAGCGCAAAGTCT
Leptin	NM_008493.3	F: TCTGAAAGATCCCACGTGCC R: AAGGCTCAGGACATTCCAGC
PPAR-α	XM_030248421.2	F: AGAGCCCCATCTGTCCTCTC R: ACTGGTAGTCTGCAAAACCAAA
PPAR-γ	XM_006505743.4	F: ATTGAGTGCCGAGTCTGTGG R: ACCTGATGGCATTGTGAGACA
TNF-α	NM_013693.3	F: ATGGCCTCCCTCTCATCAGT R: TTTGCTACGACGTGGGCTAC
GAPDH	BC023196.2	F: GAAGGTCGGTGTGAACGGAT R: AATCTCCACTTTGCCACTGC

ACC, acetyl-CoA carboxylase; ACOX-1, Acyl-CoA oxidase 1; CD68, cluster of differentiation 68; CPT-1, carnitine palmitoyltransferase-1; EMR1, epidermal growth factor-like module-containing mucin-like hormone receptor-like-1; F, forward; FAS, fatty acid synthase; GAPDH, glyceraldehyde-3-phosphate dehydrogenase; IL-1β, interleukin-1 beta; IL-6, interleukin-6; IL-10, interleukin-10; iNOS, inducible nitric oxide synthase; PPAR, peroxisome proliferator-activated receptor; R, reverse; TNF-α, tumor necrosis factor alpha.

## Data Availability

Data available on request due to restrictions eg privacy or ethical. The data presented in this study are available on request from the corresponding author.
